# Association between Dietary Intake and Lipid-Lowering Therapy: Prospective Analysis of Data from Australian Diabetes, Obesity, and Lifestyle Study (AusDiab) Using a Quantile Regression Approach

**DOI:** 10.3390/nu11081858

**Published:** 2019-08-09

**Authors:** Adelle M. Gadowski, Natalie Nanayakkara, Stephane Heritier, Dianna J. Magliano, Jonathan E. Shaw, Andrea J. Curtis, Sophia Zoungas, Alice J. Owen

**Affiliations:** 1 School of Public Health and Preventive Medicine, Monash University, Melbourne, VIC 3004, Australia; 2 Baker Heart and Diabetes Institute, Melbourne, VIC 3004, Australia

**Keywords:** food groups, vegetable, fruit, cereal, protein, dairy, lipid-lowering therapy, statins, cardiovascular disease, quantile regression

## Abstract

Lipid-lowering therapy (LLT) should be accompanied by dietary guidance for cardiovascular risk reduction; however, current evidence suggests sub-optimal dietary behaviors in those on LLT. We examined the associations between the dietary intake of key food groups (vegetables, fruit, cereal, protein, and dairy) and LLT use in Australian adults using quantile regression. We used data from the Australian Diabetes, Obesity and Lifestyle Study (AusDiab), a prospective population-based study of adults aged ≥25 years, conducted over 5 years (1999–2005). Measurements included a 121-item food frequency questionnaire and LLT use. LLT use was categorized as: LLT users (*n* = 446), commenced LLT (*n* = 565), ceased LLT (*n* = 71), and non-users (*n* = 4813). Less than 1% of the cohort met recommended intakes of all food groups at the baseline and follow up. The median daily dietary intake at the follow up among LLT users was 2.2 serves of vegetables, 1.4 serves of fruit, 2.8 serves of cereal, 2.0 serves of protein, and 1.4 serves of dairy. Adjusted analysis showed no differences across the quantiles of intake of key food groups in LLT users and commenced LLT compared to non-users. The LLT medication status is not associated with any difference in meeting recommended intakes of key foods.

## 1. Introduction

Cardiovascular diseases (CVDs) contributed to 31% or 17.9 million global deaths in 2013 [1]. The American Heart Association projects CVD to be the most expensive and prevalent killer, with associated costs expected to reach $1.1 trillion by 2035 [2]. CVD is largely preventable, and many of its risk factors, including tobacco use, high blood pressure (BP), high cholesterol, obesity, physical inactivity, and poor nutrition, are potentially modifiable [3].

The high mortality and morbidity associated with CVD make it a major target for pharmacological interventions. The complex nature of the disease has promoted a number of potential pharmacological therapies, targeting inflammation, thrombosis, oxidative stress, and cholesterol lowering. Undoubtedly, the most successful approaches to date have been therapies lowering the levels of circulating cholesterol, with statins (HMG-CoA reductase inhibitors) being the best known and most frequently used drug in those at elevated risk of CVD (high-risk primary prevention) and those with established CVD (secondary prevention) [4]. Other types of lipid-lowering agents include ezetimibe, fibrates, nicotinic acid, bile acid sequestrants, and nutraceuticals [5,6]. An estimated 2.6 million Australians (mean age 67 years) were on statin therapy in 2010–2011, which came at a cost of $1.2 billion [7]. However, adherence to statins decreases over time, with most studies reporting discontinuation rates of ≥50% [8,9,10,11,12]. Dietary patterns are strong determinants of a population’s health. Dietary patterns are associated with chronic diseases, such as CVD, hypertension, diabetes, and cancer [13,14]. Emerging evidence suggests an interplay between the effects of diet and lipid-lowering therapy (LLT) in the primary and secondary prevention of CVD. A number of population-based studies have suggested that dietary interventions increase statin-related cardio-protection [15,16]. Other studies have suggested adverse effects of LLT on dietary intake and weight, with statin users consuming a higher caloric diet and saturated fat diet compared with non-users [17]. The 2001 Lipid Management Guidelines indicate lifestyle factors, including diet, to be the first-line target intervention for those prescribed LLT [18]. One study reported an additional 19% reduction in total serum cholesterol levels with the addition of a diet and exercise program in participants on LLT [19].

However, to date, no studies have examined whether the daily dietary intake of key food groups differ between LLT users and non-users. One study suggested that LLT alone reduced the total serum cholesterol by 20%, and, with the addition of a diet and exercise program, serum cholesterol levels further decreased by 19% [19]. This is an important aspect of disease prevention, as most dietary advice given to the general population is given by referring to food groups.

Diet guidelines have been developed to reduce nutrition-related chronic diseases [20,21]. An emphasis on food-based dietary guidelines has changed the focus from single nutrients to a whole diet approach. The five food groups are: Vegetables, fruit, cereal (grain) foods, protein, and dairy. The 2003 Australian Dietary Guidelines recommend that men and women of all ages should eat five serves of vegetables, two serves of fruit, one serve of protein, and two serves of dairy per day. Women of all ages, and men aged over 60 should eat a minimum of four serves of cereal, and men aged 19–60 years should eat a minimum of six serves of cereal per day.

Studies have suggested that wide disparities exist between dietary recommendations and actual individual behaviors and indicate that compliance with the current guidelines is poor [22]. According to the most recent data from the 2011–2012 Australian Health Survey, less than 20% of adults met recommendations for vegetable, protein, and dairy intake, and approximately 40% did not meet fruit and cereal recommendations [23].

There is a paucity of data describing the association of LLT with dietary intake; understanding these interactions will provide important insights for optimal prescribing of LLT and education on recommended nutritional interventions in CVD prevention. The aim of this study was to compare adherence to guideline recommended intakes of key food groups by LLT use.

## 2. Materials and Methods 

### 2.1. Study Design and Participants

Data were obtained from the Australian Diabetes, Obesity and Lifestyle Study (AusDiab), which is a prospective population-based study of adults aged 25 years and over, across 42 randomly selected urban and rural areas across seven states and territories of Australia. A baseline assessment was conducted in 1999–2000 with 11,247 adults consenting to take part. Follow up assessments were conducted in 2004–2005, where 6400 (59.3%) of the baseline participants took part. Study methods and response rates have been published elsewhere [24]. The current study was is an analysis of data collected at the baseline (1999–2000) and follow up (2004–2005). After any missing data from participants on dietary intake and LLT status were excluded, the complete data from 5895 participants was available for analysis.

This study was conducted according to the guidelines laid down in the Declaration of Helsinki, and all procedures involving research study participants were approved by the International Diabetes Institute and the Standing Committee on Ethics in Research involving Humans, Monash University. Written informed consent was obtained from all subjects [24].

### 2.2. Outcome Measures

The outcome measures of this study were daily dietary intake (serves/d) of the five food groups. The dietary intake was collected using a validated self-administered 121-item food frequency questionnaire (FFQ), recalling intake over the previous 12 months [24]. Dietary intake was collected at the baseline (1999–2000) and follow up (2004–2005). FFQ responses of individual foods were converted into average daily intakes and individual food items were calculated in grams per day (Supplementary Table S1). The Australian Dietary Guidelines defines a standard serve of daily vegetables at approximately 75 g and a standard daily serve of fruit at 150 g. Therefore, to calculate daily vegetable and fruit serves, the total grams of daily vegetable serves was divided by 75 g and fruit by 150 g. The weight of a standard serve of cereal, dairy, and protein foods were more varied based on specific food items within these food groups. Therefore, serves for these food groups were calculated on a food item basis. Once individual grams per food item were created, foods within the same food group were added to calculate the total serves per food group.

The food groups were based on Guideline 2 of the 2003 Australian Dietary Guidelines developed by the National Health and Medical Research Council (NHMRC) [20]. The 2003 Guidelines were used in this study as these were current when the follow up data was collected in 2004–2005.

Daily dietary intake of each of the five food groups was expressed as total serves/d and classified as meeting or not meeting the Australian Dietary Guidelines [20,21].

### 2.3. Covariates

Age, sex, education, body mass index (BMI), diabetes status, history of CVD, hypertension, smoking status, and physical activity were considered as covariates. Education was categorized into two groups; up to secondary level and above secondary level. Body mass index (BMI, kg/m^2^) was calculated from height and weight measured during biomedical examinations. Hypertension was defined based on blood pressure measurements (BP ≥ 140/90 mmHg) measured by trained staff and self-reported use of medications for treating hypertension. Participants were identified to have diabetes if fasting plasma glucose was ≥7.0 mmol/L or 2 h plasma glucose was ≥11.1 mmol/L, or if participants reported using medications for diabetes. Participants were identified as having prior CVD if, at the baseline, they reported having previously had a myocardial infarction or stroke. Smoking status was self-reported, and participants were categorized as follows: Never smoker (<100 cigarettes during lifetime), former smoker (less than daily in past 3 months), and current smoker (smoked on daily basis). Physical activity was measured using the validated Active Australia Survey [25], which asks participants about the time spent engaging in moderate and vigorous physical activities during the previous week. Physical activity was categorized as sedentary (no time spent in physical acuity), insufficient (>0–149 min of moderate activity or >0–74 min of vigorous physical activity during the previous week), and sedentary (≥150 min of moderate or ≥75 min of vigorous physical activity during the previous week).

LLT use was self-reported at the baseline and follow up by participants using a self-administered questionnaire. The LLT status of participants was categorized into 4 groups LLT users, i.e., who were on LLT at the baseline and follow up; commenced LLT, i.e., participants who initiated LLT within the study period; ceased LLT, i.e., participants who ceased LLT within the study period; and non-users, participants not on LLT at the baseline or follow up. Due to insufficient numbers, regression analysis for participants who ceased LLT was not performed.

### 2.4. Statistical Analysis 

To be included in the analysis, participants had to have complete dietary and LLT data at the baseline and follow up. Participants were excluded from analyses if they had biologically implausible dietary intake based on energy intake, defined as <3300 or >17,500 kJ in men and an energy intake of <2500 kJ or >14,500 kJ in women [26]. Missing covariate data was less than 10% and not imputed.

Categorical variables were summarized as percentages and the differences between subgroups were analyzed using a χ2 test. Continuous variables were tested for normality and reported as the mean with standard deviation (SD) or as the median with interquartile range (IQR).

The descriptive analyses explored the demographic characteristics of the population sample. This was followed by bivariate analysis to establish the relationship between the independent variables and the outcome variable. Dietary intake of key food was examined at the follow up and adjusted for the baseline. Linear regression analysis was preformed to identify significant factors associated with dietary intake. Variables found to be significant (*p* < 0.05) in the univariate analysis, as well as all confounding variables of potential clinical significance, were included in the multivariable regression analysis.

Quantile regression (QR), first introduced by Koenker and Bassett in 1978, does not assume a particular parametric distribution for the response, nor does it assume a constant variance for the response, unlike least squares regression [27]. QR was adapted in the multivariate analysis to capture the full distribution of the outcome variable, as it does not assume a particular parametric distribution for the response, nor does it assume a constant variance for the response, unlike least squares regression [27]. The coefficients were estimated for the 50th quantile of the dependent variable. The 95% confidence intervals were derived from standard errors generated from 1000 bootstrap replications. A two-sided significance level of <0.05 was considered statistically significant. The coefficients for each percentile were considered to be statistically significant if their 95% CI did not cross the abscissa axis. QR was chosen, as we wanted to assess whether LLT can have an impact across the spectrum of the dietary intake. This association could not be fully addressed using linear regression, as linear regression summarized the average relationship between a list of covariates and the outcome variable based on the conditional mean function. This only provided a partial view of the relationship. By adapting the QR analysis, we were able to establish the relationship at different points in the conditional distribution of the dietary intake of each food group. QR presented a robust view of the impact of independent variables on the outcome variable; therefore, it was possible to identify the more vulnerable groups. Additionally, due to the nature of the outcome variable being highly prone to outliers, QR was more robust to non-normal errors and outliers. Adjusted QR analysis was performed for each of the five food groups. All models were adjusted for age, sex, BMI, education status, diabetes, prior CVD, hypertension, smoking status, physical activity, and baseline dietary intake per food group.

A subgroup analysis examined the association between the dietary intake at the follow up (serves/d) and the LLT status stratified by the participant’s age; <62.5 and ≥62.5 years for LLT users and <58.0 and ≥58.0 years for participants who commenced LLT. Values of age categorization were determined by the median age of participants in each LLT category. A subgroup analysis was adjusted for covariates. A QR analysis was then performed, with associations estimated at the 25th, 50th, and 75th quantile of dietary intake.

All analyses were performed using Stata statistical software version 15 (College Station, TX, USA).

## 3. Results

### 3.1. Participant Characteristics

Data from 5895 participants were analyzed. The demographic baseline characteristics of the participants, stratified by LLT status, are presented in Table 1. A total of 446 participants reported being on LLT at the baseline and follow up and were considered for this analysis to be LLT users for the course of the study. A total of 565 participants commenced LLT between the baseline and follow-up, 71 participants ceased LLT, and 4813 were non-users during the study period. The mean age of participants was highest in LLT users (62.5 years) compared to participants who ceased LLT (61.7 years), commenced LLT (57.9 years) and non-users (49.7 years) (*p* < 0.01). Over half of LLT users (52.2%) and those who commenced LLT (50.4%) were male, compared to participants who ceased LLT and non-users (46.5% and 43.9%, respectively) (*p* < 0.01). Non-users had a lower BMI (mean ± SD) (26.56 ± 4.76 kg/m^2^) compared to other groups (*p* < 0.01). The prevalence of diabetes was greatest among LLT users (50.9%) (*p* < 0.01). Prevalence of prior CVD was highest (35.0%) among LLT users, followed by those who had ceased LLT (25.4%), commenced LLT (11.7%), and non-users (3.4%) (*p* < 0.01).

### 3.2. Dietary Intake

Less than 1% of the cohort met recommended intakes of all food groups at the baseline and follow up, with no difference by LLT status (Table 2). The proportion of LLT users meeting protein intake recommendations was lower at the baseline (81.84% vs. 87.70; *p* = 0.001) and follow up compared to non-users (81.63% vs. 87.54%; *p* = 0.001). At the follow up, the median daily intake among LLT users was 2.2 serves of vegetables, 1.4 serves of fruit, 2.8 serves of cereal, 2.0 serves of protein, and 1.4 serves of dairy. The daily intake of food groups at the follow up did not significantly differ across categories of LLT use (Table 3). Non-users derived the most energy from saturated fat as a percentage of total energy 14.48% (3.44), followed by those who commenced LLT 13.75% (3.41), ceased LLT 13.07% (3.30), and LLT users 12.60% (3.23) (*p* < 0.01) (Table 1).

### 3.3. Quantile Regression

**Figure 1:**
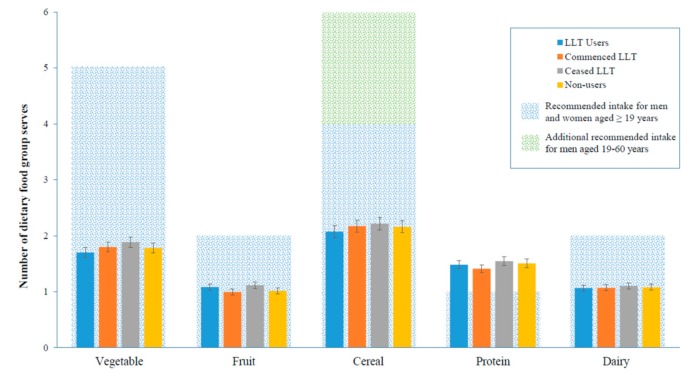
Adjusted total serves per day at the follow up of key food groups at the 25th quantile, by LLT (Lipid-lowering therapy) status, median (95% CI). Adjusted for age, gender, body mass index (kg/m^2^), smoking status, exercise status, education status, diabetes status, prior cardiovascular disease, hypertension, and baseline dietary serves per day.

**Figure 2:**
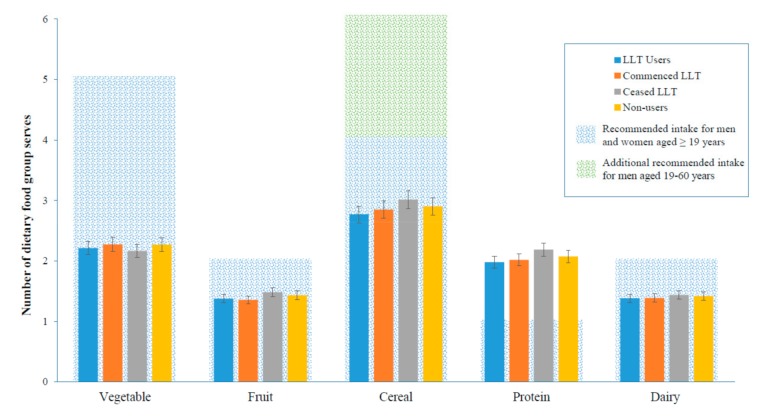
Adjusted total serves per day at the follow up of key food groups at the 50th quantile, by LLT (Lipid-lowering therapy) status, median (95% CI). Adjusted for age, gender, body mass index (kg/m^2^), smoking status, exercise status, education status, diabetes status, prior cardiovascular disease, hypertension, and baseline dietary serves per day. Lipid-lowering therapy (LLT).

**Figure 3:**
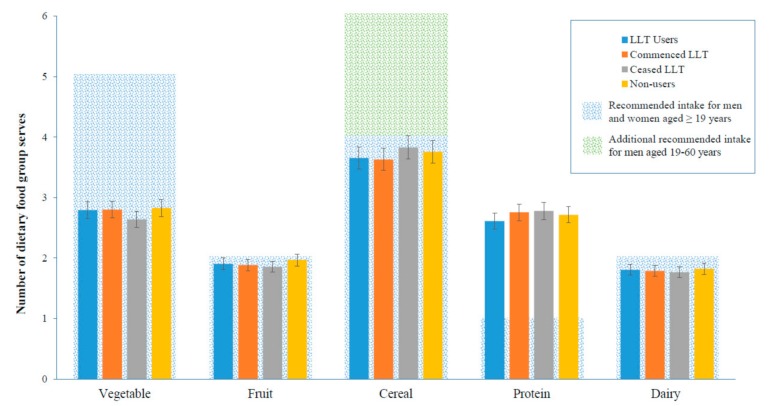
Adjusted total serves per day at the follow up of key food groups at the 75th quantile, by LLT (Lipid-lowering therapy) status, median (95% CI). Adjusted for age, gender, body mass index (kg/m^2^), smoking status, exercise status, education status, diabetes status, prior cardiovascular disease, hypertension, and baseline dietary serves per day.

There were no significant differences in the adjusted median daily intake of all key food groups in LLT users compared to non-users at the follow up (Table 4). The median dietary intake of LLT users compared to non-users did not significantly differ (Supplementary Table S2). The intake of food groups in participants who commenced LLT within the study period were not significantly different to non-users. LLT use was not significantly associated with dietary intake at the 25th (Figure 1), 50th (Figure 2), and 75th (Figure 3) quantile.

### 3.4. Subgroup Analysis

When participants were stratified based on the median age among LLT users and participants who commenced LLT within the study period, LLT users (≥62.5 years) were found to consume 0.2 fewer serves of cereal than non-users (−0.22 [−0.42, −0.02]; *p* = 0.03) (Supplementary Table S3). Participants who commenced LLT within the study period (aged ≥ 58 years) consumed 0.1 fewer serves of fruit than non-users (−0.13 [−0.24, −0.02]; *p* = 0.01) (Supplementary Table S4). No significant differences in vegetable, protein, or dairy intake were found between both LLT groups compared to non-users.

## 4. Discussion

To our knowledge, this is the first study to examine the intake of key food groups by LLT status. Despite their higher CVD risk status, participants on LLT were equally poor at meeting dietary guideline recommended intakes compared with those not on LLT. We found no significant differences in dietary intakes across all key food groups between LLT users and participants who commenced LLT within the study period compared to non-users. Our findings show a need for strategies aimed at improving dietary intake across all food groups. This is especially important for individuals who have been prescribed LLT for either primary or secondary prevention, given they are already at high risk of CVD disease. The poor quality of the diet, as evidenced by these low proportions of our study population meeting the recommended daily intake of food groups, suggest that all adults, regardless of their medication regimen, need additional education on improving their dietary intake.

Although our study is the first to examine all key food groups, there has been previous research examining individual dietary components in association with LLT. Studies examining dietary intake of vegetables, fruit, saturated fat, and caloric content by LLT use have reported inconsistent findings. The Study of Exercise and Nutrition in Older Rhode Islanders found LLT users had a significantly lower fruit and vegetable intake compared to non-users; however, this study was conducted exclusively in older and smaller sample sizes [28]. Australian [29] and Swedish [30] data has reported lower saturated fat and caloric intake associated with LLT use compared with non-users. However, American data showed saturated fat among LLT users to increase by 9.6% over a 10 year period among the general population, while the saturated fat intake of non-users remained constant [17]. LLT users may have received dietary information which causes them to eat less overall, reducing their saturated fat and overall caloric intake. However all groups in our study exceeded the recommendation that 10% of the total energy intake can be derived from saturated fat [31].

Several studies have indicated inverse associations between protein intake and blood pressure [32,33]; however few trials have reported inconsistent findings [34]. To the best of our knowledge, there is no evidence among Australian adult populations on the association between difference kinds of protein consumption (plant and animal protein) and hypertension. Therefore, we examined the relationship of protein consumption (plant and animal protein combined) and LLT.

There has been an increase in a negative perception of dairy fats, which may arise from the health promotion messages to reduce dietary saturated fat intake, hence reducing dairy intake may be the first point of action in individuals on LLT. An early study suggested a strong correlation between dairy fat and heart disease [35]. However, more recent findings have indicated that the relationship between dairy fat intake and CVD risk is unclear, with some neutral [36,37] and negative associations found [38].

These differences in findings may be due to behavioral, cultural, psychosocial, and socio-economic differences between different countries, affecting access to healthcare, dietary advice, recommendations, and uptake of preventive measures.

We found that participants who commenced LLT within the study period were no more likely to meet dietary recommendations than non-users. This finding indicates that it is unlikely for those requiring LLT to have a poorer diet profile than the general population, and also shows that those prescribed lipid-lowering therapy are not modifying their diets. This is of concern, as these people would have been prescribed LLT for primary or secondary prevention of CVD, and we would have expected them to receive dietary advice when prescribed lipid-lowering medications, ultimately increasing their intake of key food groups. However, some research has shown that people that are prescribed LLT may ignore modifiable lifestyle approaches in disease prevention as they view pharmacological approaches as a compensation for poor lifestyle choices [39]. Further research is urgently required to determine optimal education approaches to facilitate dietary change in those prescribed LLT. There is evidence to suggest that current lifestyle advice provided is insufficient to induce behavioral change to those prescribed LLT [39]. It is critical that those prescribed LLT understand that the medication is an adjunct to, and not a replacement for, dietary intake. Highlighting the importance of diet behaviors in addition to LLT, the INTERHEART study estimated that almost 90% of heart disease is caused by nine modifiable risk factors, including dietary intake [40].

Further research is also required to determine potential barriers to improve dietary behaviors and how these barriers may be overcome. Given the heightened disease risk among this population, adapting nutritional approaches to reduce the risk of developing disease is crucial.

This study has several strengths. The data consisted of a population-based sample of 5895 participants from urban, suburban, and regional areas across Australia. Participants were included in the analysis if they had baseline and follow up observations for each participant, enabling the use of robust analysis techniques. We performed QR analysis, an approach particularly suitable for examining the spectrum of outcome response in non-normally distributed data, rather than the average. This method is important from a public health and nutrient perspective, as QR allowed us to explore the characteristics associated with differences in dietary intake across LLT groups, and allowed for associations to be explored even when the mean of the outcome variable is not significantly associated with the independent variable. We also included all the relevant variables that may influence dietary behaviors, such as, age, sex, BMI, education status, medical history, including diabetes, prior CVD and hypertension status, smoking, and physical activity status.

The study design allowed us to categories participants into four pharmacological groups based on their LLT use. For those who reported using LLT at both the baseline and follow up, it was assumed that LLT was maintained between time points; however, this could not be confirmed in the data available. For all LLT user groups, we were unable to confirm the exact type, duration, and dosage of LLT therapy.

Additionally, an assumption has been made for the analyses that the self-reported dietary patterns measured represented the usual dietary intake for the study population. While FFQs for the assessment of dietary intake have limitations, they remain widely used tools in epidemiological research. Additionally, research has shown the potential for social desirability (and responding in such a way to avoid criticism) and social approval (responding to seek praise) biases in self-reported dietary data, resulting in a distortion of health related outcomes and exposures [41].

Participants were community-dwelling, so generalizability is limited to specific populations of free-living adults. Furthermore, self-reported medication use is not always reliable, due to the myriad of lipid-lowering medications and doses available on the market. We also used the 2003 Australian Dietary Guidelines as these were current in 2005 at the follow up; however, it should be noted that the baseline diet was collected in 1999–2000. As the follow up data was collected in 2004–2005, patterns of dietary intake may have changed in the intervening 15 years. Finally, our study population was predominantly from Australian community dwellings, which are English speaking, which may limit the generalizability of our findings to other populations, although not to a great extent given that global research of dietary intake among LLT users is consistent with our findings.

This study explored the associations between LLT use and key food group intake. The findings highlight the need for more effective education between patients and healthcare providers, regarding diet as a risk factor, and better support for dietary change in those receiving treatment for hyperlipidemia is required. The prescription of LLT medication should be taken in conjunction with lifestyle recommendations, including the consumption of evidence-based nutraceuticals [5]. In order to fully understand the behaviors inhibiting people from meeting the recommended daily intake of key food groups, we need to elucidate the barriers faced by individuals, especially older adults at a higher risk of disease. It is possible that issues of food security, oral health, and financial or accessibility factors are limiting the individual’s ability to meet guidelines, or some people are simply not aware of the current guidelines and do not have access to adequate dietary advice and education. It is also possible that people temporarily change their behaviors after dietary education and would benefit from repeated education to sustain optimal dietary behaviors and prevent relapse of poor habits [42].

## 5. Conclusions

In this study of 5895 Australian adults, we found there to be no difference in daily dietary intake of key food groups between LLT users and non-users. Further research is required to evaluate the impact of LLT on dietary intake, conducted in longitudinal open-label studies and placebo-controlled randomized trials. Utilizing both of these epidemiological approaches will be important to capture whether there are any ‘biological’ effects of statins on dietary intake [43] and to better understand how statin prescription influences attitudes to dietary intake. Understanding potential barriers and enablers encountered by LLT users for improving their diets will facilitate development of evidence-based strategies to achieve and sustain optimal dietary behaviors. This approach may reduce morbidity and mortality from CVD disease, providing significant social and economic benefits for individuals, families, and societies.

## Figures and Tables

**Table 1:** Baseline characteristics of study population by lipid-lowering therapy group.

Characteristic	LLT Users (*n* = 446)	Commenced LLT (*n* = 565)	Ceased LLT (*n* = 71)	Non-Users (*n* = 4813)	*p* Value
Age, mean (SD)	62.54 (9.29) ^a^	57.92 (10.20) ^bc^	61.72 (11.0) ^ac^	49.67 (12.34) ^d^	<0.01
Sex, *n* (%)					
Male	233 (52.2) ^a^	313 (50.4) ^b^	33 (46.5) ^abc^	2113 (43.9) ^c^	<0.01
Education, *n* (%)					
Higher than secondary level education	131 (29.4) ^a^	195 (34.5) ^b^	21 (29.6) ^c^	2073 (43.1) ^d^	<0.01
BMI kg/m^2^, mean (SD)	28.46 (4.73) ^a^	28.03 (4.75) ^a^	28.46 (4.67) ^a^	26.56 (4.76) ^b^	<0.01
Diabetes, *n* (%)					
Yes	227 (50.9) ^a^	219 (38.8) ^b^	32 (45.1) ^abc^	987 (20.5) ^d^	<0.01
Prior CVD, *n* (%)					
Yes	156 (35.0) ^a^	66 (11.7) ^b^	18 (25.4) ^ac^	163 (3.4) ^d^	<0.01
Hypertension, *n* (%)					
Hypertensive	286 (64.1) ^a^	306 (54.2) ^b^	43 (60.6) ^abc^	1198 (24.9) ^d^	<0.01
Smoking status, *n* (%)					
Never smoker	227 (52.0) ^a^	299 (53.4) ^b^	44 (62.9) ^c^	2863 (60.3) ^c^	<0.01
Former smoker	165 (37.8)	200 (35.7)	18 (25.7)	1353 (28.5)	
Current smoker	45 (10.3)	61 (10.9)	8 (11.4)	533 (11.2)	
Physical activity, *n* (%)					
Sedentary	73 (16.4)	97 (17.2)	15 (21.1)	733 (15.3)	0.675
Insufficient	133 (29.9)	172 (30.5)	23 (32.4)	1474 (30.7)	
Sufficient	239 (53.7)	295 (50.3)	33 (46.5)	2596 (54.1)	
Energy intake, kJ/day					
	7430.70 (2483.87) ^a^	7838.61 (2548.32) ^b^	7351.24 (2618.71) ^b^	8018.08 (2735.2) ^c^	0.01
Saturated fat as proportion energy intake, % (SD)					
	12.60 (3.23) ^a^	13.75 (3.41) ^ab^	13.07 (3.30) ^ab^	14.48 (3.44) ^c^	<0.01

Body mass index (BMI); blood pressure (BP); cardiovascular disease (CVD); lipid-lowering therapy (LLT); standard deviation (SD). Difference in superscript letters indicate a significant difference (*p* < 0.05). Hypertension: Hypertensive >140/90 BP or on tablets for hypertension, normal blood pressure ≤140/90 mmHg. Physical activity: Sedentary; 0 min of physical activity per week, insufficient; >0–149 min of physical activity per week, sufficient; ≥150 min of moderate or ≥75 min of vigorous physical activity during the previous week. Missing values: Education 1, body mass index 28, hypertension 6, smoking status 79, physical activity 10. Continuous variables were first analyzed by two-way ANOVA and Bonferroni post hoc tests. Significance between categories was determined chi-square tests.

**Table 2:** Baseline characteristics of study population by lipid-lowering therapy group.

	LLT Users (*n* = 446)	Commenced LLT (*n* = 565)	Ceased LLT (*n* = 71)	Non LLT Users (*n* = 4813)	*p* Value
**Proportion meeting guidelines at baseline, *n* (%)**					
All food groups	2 (0.45)	1 (0.18)	0 (0.00)	7 (0.15)	0.506
Vegetables	13 (2.91)	15 (2.65)	2 (2.82)	115 (2.39)	0.894
Fruit	126 (28.25)	160 (28.32)	19 (26.76)	1249 (25.95)	0.501
Cereal	109 (24.44)	117 (20.71)	14 (19.72)	902 (18.74)	0.027
Protein	365 (81.84) ^a^	474 (83.89) ^ab^	63 (88.73) ^ab^	4221 (87.70) ^b^	0.001
Dairy	90 (20.18)	109 (19.29)	14 (19.72)	1009 (20.96)	0.802
**Proportion meeting guidelines at year 5, *n* (%)**					
All food groups	1 (0.23)	0 (0.00)	0 (0.00)	3 (0.06)	0.560
Vegetable	6 (1.36)	10 (1.79)	1 (1.45)	95 (2.00)	0.797
Fruit	116 (26.30)	148 (26.48)	20 (28.99)	1354 (28.49)	0.613
Cereal	75 (17.01)	74 (13.24)	6 (8.70)	668 (14.05)	0.166
Protein	360 (81.63) ^a^	468 (83.72) ^ab^	61 (88.41) ^ab^	4161 (87.54) ^b^	0.001
Dairy	85 (19.27)	103 (18.43)	12 (17.39)	949 (19.97)	0.786

Lipid-lowering therapy (LLT). Significance between categorical variables was determined by chi-square tests. Difference in superscript letters indicate a significant difference (*p* < 0.05).

**Table 3:** Adjusted total serves per day at follow up of key food groups by LLT status, median (95% CI).

Food Group	LLT Users (*n* = 446)	Commenced LLT (*n* = 565)	Ceased LLT (*n* = 71)	Non LLT Users (*n* = 4813)
Vegetable	2.21 (2.11, 2.31)	2.27 (2.20, 2.35)	2.16 (2.03, 2.30)	2.27 (2.24, 2.30)
Fruit	1.38 (1.31, 1.45)	1.35 (1.28, 1.42)	1.48 (1.34, 1.62)	1.43 (1.41, 1.46)
Cereal	2.77 (2.63, 2.91)	2.85 (2.74, 2.96)	3.01 (2.77, 3.26)	2.90 (2.86, 2.95)
Protein	1.98 (1.88, 2.08)	2.02 (1.93, 2.11)	2.19 (1.96, 2.42)	2.08 (2.04, 2.11)
Dairy	1.38 (1.31, 1.46)	1.39 (1.33, 1.44)	1.44 (1.27, 1.60)	1.42 (1.40, 1.44)

Lipid-lowering (LLT) therapy. Adjusted for age, gender, body mass index (kg/m^2^), smoking status, exercise status, education status, diabetes status, prior cardiovascular disease, hypertension, and baseline dietary serves per day. LLT categories: LLT users, i.e., who were on LLT at the baseline and follow up; commenced LLT, i.e., participants who initiated LLT within the study period; ceased LLT, i.e., participants who ceased LLT within the study period; and non-users, participants not on LLT at the baseline or follow up. There were no significant differences between groups.

**Table 4:** Adjusted median (95% CI) intake at the 50th quantile for food group consumption at the follow up (serves/day) among LLT users and commenced LLT users, compared to non-LLT users.

Food Group	LLT Users (*n* = 446)	Non-Users (Ref.)	Commenced LLT (*n* = 565)	Non-Users (Ref.)
Vegetable	−0.09 (−0.20, 0.02)		−0.0001 (−0.08, 0.08)	
Fruit	−0.04 (−0.12, 0.04)		−0.07 (−0.14, 0.001)	
Cereal	−0.13 (−0.28, 0.02)		−0.07 (−0.19, 0.05)	
Protein	−0.09 (−0.21, 0.03)		−0.06 (−0.15, 0.04)	
Dairy	−0.03 (−0.11, 0.04)		−0.03 (−0.09, 0.03)	

Lipid-lowering therapy (LLT). Adjusted for age, gender, body mass index (kg/m^2^), smoking status, exercise status, education status, diabetes status, prior cardiovascular disease, hypertension, and baseline dietary serves per day. LLT categories: LLT users, i.e. who were on LLT at the baseline and follow up; non-users, participants not on LLT at the baseline or follow up. There were no significant differences between groups.

## Supplementary Materials

The following are available online at https://www.mdpi.com/2072-6643/11/8/1858/s1. Table S1: Food group composition. Table S2: Difference in adjusted median (95% CI) in food group consumption (serves/day) at follow up among lipid-lowering users and participants who commenced lipid-lowering therapy, compared to non- lipid-lowering users. Table S3: Difference in adjusted median (95% CI) intake in food group consumption (serves/day) at follow up among lipid-lowering users, compared to non-users, stratified by participants aged <62.5 and ≥62.5 years. Table S4: Difference in adjusted median (95% CI) intake in food group consumption (serves/day) at follow up among users who commenced lipid-lowering, compared to non-users, stratified by participants aged <58 and ≥58 years.

## References

[1] Abubakar I., Tillmann T., Banerjee A. (2015). Global, regional, and national age-sex specific all-cause and cause-specific mortality for 240 causes of death, 1990–2013: A systematic analysis for the global burden of disease study 2013. Lancet.

[2] American Heart Association (2017). Cardiovascular Disease: A Costly Burden for America Projections through 2035.

[3] Yusuf S., Reddy S., Ôunpuu S., Anand S. (2001). Global burden of cardiovascular diseases: Part I: General considerations, the epidemiologic transition, risk factors, and impact of urbanization. Circulation.

[4] Megson I.L., Whitfield P.D., Zabetakis I. (2016). Lipids and cardiovascular disease: Where does dietary intervention sit alongside statin therapy?. Food Funct..

[5] Poli A., Visioli F. (2019). Pharmacology of nutraceuticals with lipid lowering properties. High Blood Press. Cardiovasc. Prev..

[6] Cicero A.F., Colletti A., Bajraktari G., Descamps O., Djuric D.M., Ezhov M., Fras Z., Katsiki N., Langlois M., Latkovskis G. (2017). Lipid-lowering nutraceuticals in clinical practice: Position paper from an international lipid expert panel. Nutr. Rev..

[7] Hamilton-Craig I.R. (2014). Prescribing statins: The real issues. Med. J. Aust..

[8] Simons L.A., Levis G., Simons J. (1996). Apparent discontinuation rates in patients prescribed lipid-lowering drugs. Med. J. Aust..

[9] Ellis J.J., Erickson S.R., Stevenson J.G., Bernstein S.J., Stiles R.A., Fendrick A.M. (2004). Suboptimal statin adherence and discontinuation in primary and secondary prevention populations. J. Gen. Intern. Med..

[10] Deambrosis P., Saramin C., Terrazzani G., Scaldaferri L., Debetto P., Giusti P., Chinellato A. (2007). Evaluation of the prescription and utilization patterns of statins in an italian local health unit during the period 1994–2003. Eur. J. Clin. Pharmacol..

[11] Vinker S., Shani M., Baevsky T., Elhayany A. (2008). Adherence with statins over 8 years in a usual care setting. Am. J. Manag. Care.

[12] Ofori-Asenso R., Jakhu A., Zomer E., Curtis A.J., Korhonen M.J., Nelson M., Gambhir M., Tonkin A., Liew D., Zoungas S. (2017). Adherence and persistence among statin users aged 65 years and over: A systematic review and meta-analysis. J. Gerontol. A Biol. Sci. Med. Sci..

[13] Hu F.B., Rimm E.B., Stampfer M.J., Ascherio A., Spiegelman D., Willett W.C. (2000). Prospective study of major dietary patterns and risk of coronary heart disease in men. Am. J. Clin. Nutr..

[14] Jacques P.F., Tucker K.L. (2001). Are dietary patterns useful for understanding the role of diet in chronic disease?. Am. J. Clin. Nutr..

[15] Panagiotakos D.B., Georgousopoulou E.N., Georgiopoulos G.A., Pitsavos C., Chrysohoou C., Skoumas I., Ntertimani M., Laskaris A., Papadimitriou L., Tousoulis D. (2015). Adherence to mediterranean diet offers an additive protection over the use of statin therapy: Results from the attica study (2002–2012). Curr. Vasc. Pharmacol..

[16] Wang H., Blumberg J.B., Chen C.Y.O., Choi S.W., Corcoran M.P., Harris S.S., Jacques P.F., Kristo A.S., Lai C.Q., Lamon-Fava S. (2014). Dietary modulators of statin efficacy in cardiovascular disease and cognition. Mol. Asp. Med..

[17] Sugiyama T., Tsugawa Y., Tseng C.H., Kobayashi Y., Shapiro M.F. (2014). Is there gluttony in the time of statins? Different time trends of caloric and fat intake between statin-users and non-users among us adults. JAMA Intern. Med..

[18] Barter P., Best J., Boyden A., Cooper C., Gillam I., Mansfield P. (2001). Lipid management guidelines—2001. Med. J. Aust..

[19] Barnard R.J., DiLauro S.C., Inkeles S.B. (1997). Effects of intensive diet and exercise intervention in patients taking cholesterol-lowering drugs. Am. J. Cardiol..

[20] National Health and Medical Research Council (2003). Dietary Guidelines for Australian Adults.

[21] National Health and Medical Research Council (1999). Dietary Guidelines for Older Australians.

[22] Australian Institute of Health and Welfare 2014 (2014). Australia’s Health 2014.

[23] Australian Bureau of Statistics (2012). Australian Health Survey: First Results, 2011–2012.

[24] Dunstan D.W., Zimmet P.Z., Welborn T.A., Cameron A.J., Shaw J., De Courten M., Jolley D., McCarty D.J., Committee A.S. (2002). The Australian Diabetes, Obesity and Lifestyle Study (AusDiab)—Methods and response rates. Diabetes Res. Clin. Pract..

[25] Australian Institute of Health and Welfare (2003). The Active Australia Survey: A Guide and Manual for Implementation, Analysis and Reporting.

[26] Subar A.F., Thompson F.E., Kipnis V., Midthune D., Hurwitz P., McNutt S., McIntosh A., Rosenfeld S. (2001). Comparative validation of the block, willett, and national cancer institute food frequency questionnaires: The eating at America’s table study. Am. J. Epidemiol..

[27] Koenker R., Bassett G. (1978). Regression quantiles. Econometrica.

[28] Lofgren I., Greene G., Schembre S., Delmonico M.J., Riebe D., Clark P. (2010). Comparison of diet quality, physical activity and biochemical values of older adults either reporting or not reporting use of lipid-lowering medication. J. Nutr. Health Aging.

[29] Johal S., Jamsen K.M., Bell J.S., Mc Namara K.P., Magliano D.J., Liew D., Ryan-Atwood T.E., Anderson C., Ilomäki J. (2017). Do statin users adhere to a healthy diet and lifestyle? The Australian diabetes, obesity and lifestyle study. Eur. J. Prev. Cardiol..

[30] Lytsy P., Burell G., Westerling R. (2012). Cardiovascular risk factor assessments and health behaviours in patients using statins compared to a non-treated population. Int. J. Behav. Med..

[31] Van Horn L., McCoin M., Kris-Etherton P.M., Burke F., Carson J.A.S., Champagne C.M., Karmally W., Sikand G. (2008). The evidence for dietary prevention and treatment of cardiovascular disease. J. Am. Diet. Assoc..

[32] Blumenthal J.A., Babyak M.A., Hinderliter A., Watkins L.L., Craighead L., Lin P.H., Caccia C., Johnson J., Waugh R., Sherwood A. (2010). Effects of the dash diet alone and in combination with exercise and weight loss on blood pressure and cardiovascular biomarkers in men and women with high blood pressure: The encore study. Arch. Intern. Med..

[33] Liu L., Ikeda K., Sullivan D.H., Ling W., Yamori Y. (2002). Epidemiological evidence of the association between dietary protein intake and blood pressure: A meta-analysis of published data. Hypertens. Res..

[34] Liu Z.M., Ho S.C., Chen Y.M., Woo J. (2013). Effect of soy protein and isoflavones on blood pressure and endothelial cytokines: A 6-month randomized controlled trial among postmenopausal women. J. Hypertens..

[35] Turpeinen O. (1979). Effect of cholesterol-lowering diet on mortality from coronary heart disease and other causes. Circulation.

[36] Guo J., Astrup A., Lovegrove J.A., Gijsbers L., Givens D.I., Soedamah-Muthu S.S. (2017). Milk and dairy consumption and risk of cardiovascular diseases and all-cause mortality: Dose–response meta-analysis of prospective cohort studies. Eur. J. Epidemiol..

[37] Goldbohm R.A., Chorus A.M., Galindo Garre F., Schouten L.J., van den Brandt P.A. (2011). Dairy consumption and 10-y total and cardiovascular mortality: A prospective cohort study in the Netherlands. Am. J. Clin. Nutr..

[38] Alexander D.D., Bylsma L.C., Vargas A.J., Cohen S.S., Doucette A., Mohamed M., Irvin S.R., Miller P.E., Watson H., Fryzek J.P. (2016). Dairy consumption and CVD: A systematic review and meta-analysis. Br. J. Nutr..

[39] McAleer S., Cupples M., Neville C., McKinley M., Woodside J., Tully M. (2016). Statin prescription initiation and lifestyle behaviour: A primary care cohort study. BMC Fam. Pract..

[40] Buttar H.S., Li T., Ravi N. (2005). Prevention of cardiovascular diseases: Role of exercise, dietary interventions, obesity and smoking cessation. Exp. Clin. Cardiol..

[41] Hebert J.R., Ma Y., Clemow L., Ockene I.S., Saperia G., Stanek E.J., Merriam P.A., Ockene J.K. (1997). Gender differences in social desirability and social approval bias in dietary self-report. Am. J. Epidemiol..

[42] Reeves R.S., Foreyt J.P., Scott L.W., Mitchell R.E., Wohlleb J., Gotto A. (1983). Effects of a low cholesterol eating plan on plasma lipids: Results of a three-year community study. Am. J. Public Health.

[43] Jula A., Marniemi J., Huupponen R., Virtanen A., Rastas M., Rönnemaa T. (2002). Effects of diet and simvastatin on serum lipids, insulin, and antioxidants in hypercholesterolemic men: A randomized controlled trial. JAMA.

