# Supplementary material for: Association between Dietary Intake and Lipid-Lowering Therapy: Prospective Analysis of Data from Australian Diabetes, Obesity, and Lifestyle Study (AusDiab) Using a Quantile Regression Approach
Source: Nutrients. 2019 Aug 9;11(8):1858. doi: 10.3390/nu11081858 (PMC6724025; doi:10.3390/nu11081858)
Supplement: Supplementary file 1 [file nutrients-11-01858-s001.pdf]

Supplementary Table S1 Food group composition

Food group	Source	Serving size (g)
**Vegetables**		
	Tomato sauce	75
	Onion	
	Garlic	
	Potatoes	
	Tomatoes	
	Capsicum	
	Lettuce	
	Cucumber	
	Celery	
	Beetroot	
	Carrots	
	Cabbage	
	Cauliflower	
	Broccoli	
	Spinach	
	Peas	
	Green Beans	
	Bean sprouts	
	Baked beans	
	Tofu	
	Other beans	
	Pumpkin	
	Mushroom	
	Zucchini	
**Fruit**		
	Tinned fruit	150
	Orange	
	Apple	
	Pear	
	Banana	
	Melon	
	Pineapple	
	Strawberry	
	Apricot	
	Peach/nectarine	
	Mango	
	Avocado	
**Cereal**		
	Hi fibre white bread	80
	White bread	
	Wholemeal bread	
	Rye bread	
	Multigrain bread	
	All bran	30
	Bran flakes	
	Weet-bix	
	Corn flakes	
	Muesli	
	Porridge	120
	Rice	150
	Pasta	
	Crackers	35
**Protein**		
	Egg	120
	Nuts	30
	Beef	65
	Veal	
	Lamb	
	Pork	
	Chicken	80
	Fish	100
	Fried fish	

	Tinned fish	95
**Dairy**		
	Full cream milk	250
	Reduced fat milk	
	Skim milk	
	Soy milk	
	Hard cheese	40
	Soft cheese	
	Cream cheese	
	Ricotta cheese	120
	Cottage cheese	
	Yoghurt	200

1

Supplementary Table S2 Difference in adjusted median (95% CI) in food group consumption (serves/day) at follow up among lipid-lowering users and participants who commenced lipid-lowering therapy, compared to non- lipid-lowering users.

	Quantile Coefficient. (95 % CI)														
	Vegetable			Fruit			Cereal			Protein			Dairy		
Explanatory Variable	Q25	Q50	Q75	Q25	Q50	Q75	Q25	Q50	Q75	Q25	Q50	Q75	Q25	Q50	Q75
Age	0.003 (0.001, 0.006)*	0.002 (-0.0003, 0.005)	0.001202 (-0.002, 0.006)	0.004 (0.002, 0.006)**	0.006 (0.004, 0.008)**	0.005 (0.001, 0.008)**	0.003 (0.002, 0.006)*	0.003 (-0.001, 0.007)	0.001 (-0.003, 0.006)	-0.005 (-0.008, -0.003)**	-0.006 (-0.009, -0.004)**	-0.008 (-0.012, -0.004)**	0.001 (0.00002, 0.002)*	0.003 (0.001, 0.004)**	0.002 (-0.00002, 0.005)
Sex															
Male (Ref.)															
Female	0.093 (0.043, 0.141)**	0.046 (-0.009, 0.101)	0.015 (-0.065, 0.95)	0.021 (-0.018, 0.061)	0.049 (0.006, 0.091)*	0.012 (-0.057, 0.082)	-0.277 (-0.353, -0.200)**	-0.355 (-0.450, -0.261)**	-0.389 (-0.494, -0.285)**	-0.146 (-0.207, -0.087)**	-0.213 (-0.278, -0.148)**	-0.256 (-0.357, -0.156)**	0.016 (-0.005, 0.036)	0.061 (0.018, 0.104)**	0.059 (0.009, 0.110)*
BMI kg/m^2	0.003 (-0.002, 0.009)	0.004 (-0.001, 0.010)	0.006 (-0.002, 0.015)	0.001 (-0.003, 0.006)	0.005 (0.001, 0.010)*	0.009 (0.001, 0.017)*	-0.008 (-0.016, -0.00004)*	-0.003 (-0.011, 0.005)	-0.009 (-0.021, 0.003)	0.009 (0.003, 0.016)**	0.010 (0.004, 0.017)**	0.015 (0.004, 0.025)**	0.001 (-0.002, 0.003)	-0.001 (-0.005, 0.004)	0.005 (-0.0004, 0.011)
Education															
Secondary level or lower (Ref.)															
Higher than secondary level	0.011 (-0.043, 0.065)	0.021 (-0.034, 0.076)	-0.025 (-0.096, 0.047)	0.029 (-0.007, 0.065)	0.040 (-0.007, 0.086)	0.015 (-0.058, 0.089)	0.029 (-0.052, 0.109)	0.011 (-0.069, 0.090)	0.009 (-0.096, 0.113)	0.009 (-0.047, 0.065)	-0.020 (-0.077, 0.038)	-0.095 (-0.189, -0.001)*	0.017 (-0.004, 0.037)	0.027 (-0.014, 0.067)	0.006 (-0.049, 0.061)
Diabetes															
Yes	0.039 (-0.043, 0.065)	0.030 (-0.042, 0.102)	-0.020 (-0.115, 0.075)	-0.010 (-0.057, 0.038)	0.0003 (-0.063, 0.063)	0.42 (-0.044, 0.129)	0.043 (-0.048, 0.134)	0.033 (-0.074, 0.141)	0.148 (0.019, 0.277)	-0.027 (-0.096, 0.043)	-0.031 (-0.097, 0.034)	0.007 (-0.122, 0.137)	-0.005 (-0.027, 0.016)	-0.012 (-0.061, 0.036)	0.001 (-0.068, 0.070)
No (Ref.)															
Prior CVD															
Yes	-0.059 (-0.043, 0.065)	-0.052 (-0.179, 0.075)	-0.124 (-0.302, 0.055)	-0.066 (-0.169, 0.036)	-0.0121 (-0.207, -0.035)**	-0.195 (-0.241, -0.049)**	-0.096 (-0.267, 0.074)	-0.084 (-0.267, 0.100)	-0.077 (-0.307, 0.152)	-0.214 (-0.329, -0.098)**	-0.136 (-0.269, -0.004)*	-0.144 (-0.352, 0.063)	-0.008 (-0.059, 0.043)	-0.003 (-0.077, 0.071)	0.062 (-0.030, 0.154)
No (Ref.)															
Hypertension mmHg															
Hypertensive	-0.003 (-0.043, 0.058)	0.029 (-0.043, 0.102)	0.041 (-0.055, 0.136)	0.015 (-0.038, 0.067)	0.044 (-0.008, 0.097)	0.079 (-0.006, 0.164)	0.001 (-0.090, 0.092)	-0.064 (-0.162, 0.034)	-0.014 (-0.136, 0.108)	-0.011 (-0.084, 0.062)	-0.005 (-0.081, 0.071)	-0.005 (-0.135, 0.124)	-0.003 (-0.025, 0.020)	0.040 (-0.007, 0.086)	0.014 (-0.051, 0.079)
Normal BP (Ref.)															
Smoking status															
Never smoker (Ref.)															
Former smoker	-0.060 (-0.114, -0.006)*	-0.081 (-0.138, -0.023)**	-0.064 (-0.146, 0.018)	-0.069 (-0.112, -0.025)**	-0.055 (-0.104, -0.006)*	-0.065 (-0.144, 0.014)	-0.008 (-0.094, 0.078)	-0.048 (-0.134, 0.038)	-0.023 (-0.141, 0.095)	-0.009 (-0.061, 0.042)	-0.001 (-0.061, 0.058)	0.006 (-0.095, 0.107)	-0.011 (-0.034, 0.012)	-0.017 (-0.060, 0.027)	-0.012 (-0.067, 0.043)
Current smoker	-0.119 (-0.226, -0.011)*	-0.004 (-0.105, 0.097)	0.027 (-0.097, 0.151)	-0.120 (-0.166, -0.075)**	-0.145 (-0.206, -0.084)**	-0.229 (-0.339, -0.119)**	-0.201 (-0.316, -0.085)**	-0.223 (-0.365, -0.081)**	-0.076 (-0.259, 0.106)	0.023 (-0.096, 0.142)	0.108 (-0.005, 0.221)	0.201 (0.050, 0.353)**	-0.053 (-0.129, 0.023)	-0.084 (-0.161, -0.007)*	-0.024 (-0.135, 0.188)
Physical activity															
Sedentary	-0.062 (-0.147, 0.022)	-0.057 (-0.127, 0.12)	0.085 (-0.016, 0.185)	-0.092 (-0.142, -0.042)**	-0.142 (-0.193, -0.090)**	-0.291 (-0.387, -0.195)**	-0.050 (-0.142, 0.043)	-0.038 (-0.151, 0.076)	0.093 (0.065, 0.251)*	-0.048 (-0.126, 0.030)	-0.077 (-0.157, 0.003)	-0.071 (-0.189, 0.047)	-0.016 (-0.045, 0.013)	-0.054 (-0.111, 0.004)	-0.007 (-0.081, 0.068)

Explanatory Variable	Quantile Coefficient. (95 % CI)														
	Vegetable			Fruit			Cereal			Protein			Dairy		
	Q25	Q50	Q75	Q25	Q50	Q75	Q25	Q50	Q75	Q25	Q50	Q75	Q25	Q50	Q75
Insufficient	0.011 (-0.043, 0.064)	-0.004 (-0.072, 0.063)	0.051 (-0.026, 0.128)	-0.010 (-0.053, 0.033)	-0.029 (-0.078, 0.021)	-0.068 (-0.152, 0.015)	0.036 (-0.043, 0.116)	0.033 (-0.051, 0.117)	0.094 (-0.016, 0.204)	0.017 (-0.039, 0.074)	-0.049 (-0.111, 0.013)	-0.039 (-0.134, 0.057)	0.006 (-0.014, 0.027)	0.021 (-0.020, 0.060)	-0.157 (-0.073, 0.041)
Sufficient (Ref.)															
LLT															
Yes	-0.090 (-0.178, -0.001)*	-0.090 (-0.198, 0.018)	-0.040 (-0.201, 0.120)	0.074 (-0.005, 0.153)	-0.039 (-0.118, 0.040)	-0.040 (-0.164, 0.084)	-0.088 (-0.205, 0.029)	-0.133 (-0.284, 0.018)	-0.099 (-0.293, 0.095)	-0.026 (-0.124, 0.072)	-0.091 (-0.210, 0.029)	-0.043 (-0.237, 0.152)	-0.012 (-0.049, 0.025)	-0.033 (-0.110, 0.044)	-0.029 (-0.113, 0.064)
No (Ref.)															

BMI, body mass index; BP, blood pressure; CVD, cardiovascular disease; LLT, lipid lowering therapy

LLT categories: LLT users, i.e. who were on LLT at baseline and follow up; commenced LLT, i.e. participants who initiated LLT within the study period; ceased LLT, i.e. participants who ceased LLT within the study period and non-users, participants not on LLT at baseline or follow up.

Hypertension: hypertensive >140/90 BP or on tablets for hypertension, normal blood pressure ≤ 140/90 mmHg.

Physical activity: sedentary; 0 minutes of physical activity per week, insufficient; 0 – 150 minutes physical activity per week, sufficient; over 150 minutes physical activity per week.

* Significance at p <0.05, ** significance at p <0.01

Supplementary Table S3 Difference in adjusted median (95% CI) intake in food group consumption (serves/day) at follow up among lipid-lowering users, compared to non-users, stratified by participants aged <62.5 and ≥62.5 years

Food group	Quantile	Quantile Coefficient. (95 % CI)			
		Age <62.5 (n= 211)	Non-users (n= 4034) (Ref.)	Age ≥62.5 (n= 235)	Non-users (n= 779) (Ref.)
Vegetable					
	Q25	-0.08 (-0.20, 0.03)		-0.08 (-0.22, 0.07)	
	Q50	-0.14 (-0.31, 0.02)		0.02 (-0.13, 0.17)	
	Q75	0.01 (-0.17, 0.20)		-0.16 (-0.38, 0.06)	
Fruit					
	Q25	0.05 (-0.06, 0.15)		0.05 (-0.07, 0.18)	
	Q50	-0.01 (-0.13, 0.11)		-0.05 (-0.16, 0.05)	
	Q75	-0.04 (-0.20, 0.12)		0.03 (-0.15, 0.22)	
Cereal					
	Q25	-0.02 (-0.19, 0.14)		-0.09 (-0.28, 0.11)	
	Q50	-0.01 (-0.23, 0.21)		-0.22 (-0.42, -0.02)*	
	Q75	-0.08 (-0.34, 0.18)		-0.20 (-0.53, 0.12)	
Protein					
	Q25	-0.08 (-0.24, 0.08)		0.03 (-0.09, 0.15)	
	Q50	-0.14 (-0.27, 0.002)		-0.05 (-0.25, 0.15)	
	Q75	-0.17 (-0.41, 0.07)		0.15 (-0.09, 0.38)	
Dairy					
	Q25	-0.04 (-0.11, 0.03)		-0.01 (-0.11, 0.08)	
	Q50	-0.09 (-0.18, 0.002)		0.01 (-0.10, 0.12)	
	Q75	-0.11 (-0.27, 0.05)		0.03 (-0.12, 0.18)	

Adjusted for gender, body mass index (kg/m^2), smoking status, exercise status, education status, diabetes status, prior cardiovascular disease, hypertension and baseline dietary serves per day.
* Significance at p <0.05, ** significance at p <0.01

Supplementary Table S4 Difference in adjusted median (95% CI) intake in food group consumption (serves/day) at follow up among users who commenced lipid-lowering, compared to non-users, stratified by participants aged <58 and ≥58 years

Food group	Quantile	Quantile Coefficient. (95 % CI)			
		Age <58 (n= 281)	Non-users (n= 3631) (Ref.)	Age ≥58 (n= 284)	Non-users (n= 1182) (Ref.)
Vegetable					
	Q25	0.07 (-0.06, 0.19)		0.04 (-0.08, 0.16)	
	Q50	0.05 (-0.06, 0.16)		-0.001 (-0.12, 0.11)	
	Q75	0.06 (-0.11, 0.23)		-0.04 (-0.24, 0.15)	
Fruit					
	Q25	0.003 (-0.07, 0.08)		-0.06 (-0.17, 0.05)	
	Q50	-0.02 (-0.13, 0.08)		-0.13 (-0.24, -0.02)*	
	Q75	0.01 (-0.19, 0.21)		-0.15 (-0.30, 0.0001)	
Cereal					
	Q25	0.06 (-0.06, 0.18)		-0.09 (-0.30, 0.13)	
	Q50	-0.04 (-.21, 0.13)		-0.06 (-0.22, 0.11)	
	Q75	-0.10 (-0.29, 0.09)		-0.14 (-0.38, 0.09)	
Protein					
	Q25	-0.16 (-0.29, -0.02)*		-0.04 (-0.15, 0.06)	
	Q50	-0.05 (-0.18, 0.08)		-0.04 (-0.17, 0.10)	
	Q75	0.001 (-0.24, 0.25)		0.08 (-0.12, 0.27)	
Dairy					

Q25	-0.02 (-0.06, 0.03)	-0.05 (-0.14, 0.04)
Q50	-0.03 (-0.11, 0.05)	-0.02 (-0.10, 0.07)
Q75	-0.01 (-0.13, 0.12)	-0.01 (-0.11, 0.10)

Adjusted for gender, body mass index (kg/m^2), smoking status, exercise status, education status, diabetes status, prior cardiovascular disease, hypertension and baseline dietary serves per day.

* Significance at $p < 0.05$, ** significance at $p < 0.01$
